# Molecular identification and genetic variation of *Alternaria *species isolated from tomatoes using ITS1 sequencing and inter simple sequence repeat methods

**DOI:** 10.18502/cmm.5.2.1154

**Published:** 2019-06

**Authors:** Abdelnasser Mohammadi, Seifollah Bahramikia

**Affiliations:** 1 Department of Biology, Faculty of Science, Lorestan University, Khorramabad, Iran

**Keywords:** Alternaria alternata, Inter simple sequence repeat methods, ITS rRNA, Tomato

## Abstract

**Background and Purpose:**

*Alternaria* is one of the most abundant fungi that exists in numerous places around the world. This saprophytic fungus causes diseases in plants and accounts for the spoilage of cereals in warehouses. The aim of this study was to identify *Alternaria* isolates based on their morphological characteristics and internal transcribed spacer ribosomal RNA (ITS rRNA) sequencing method. To this end, genetic diversity in the isolates was also examined using inter simple sequence repeat (ISSR) markers.

**Materials and Methods:**

To conduct this research, a total of 60 tomato samples with black spots were collected from supermarkets in Khorramabad City, Iran, in the winter of 2017. The specimens were cultured on a potato dextrose agar medium. After the purification of the fungus by the single-spore method, the identification of the species was carried out using morphological characteristics and ITS rRNA sequencing by polymerase chain reaction. The genetic diversity of the identified species with four primers was evaluated using the ISSR marker.

**Results:**

Based on the sequencing of the ITS1 region, all the isolates were identified as *A. alternata*. Cluster diagrams for the ISSR marker were classified into six distinct groups. The mean polymorphism information content was obtained as 0.35, indicating the effectiveness of the primers in the separation of the isolates.

**Conclusion:**

The sequencing of ITS1 led to the identification of *Alternaria* species that are morphologically similar. The production of various mycotoxins by *A. Alternata* species leads to the contamination of livestock and human food. Regarding this, the investigation of the genetic diversity of *A. alternata* species using the ISSR marker would facilitate the identification of suitable and effective strategies for controlling the fungal and mycotoxin contamination of human nutrition.

## Introduction

One of the major causes of food contamination is the presence of fungal spores in the air. Fungi not only cause high economic losses but also expose the human and animals’ health to high risk by producing mycotoxin [1-3]. *Alternaria* are ubiquitous filamentous fungi widely distributed in the environment. The spores of this genus can be separated from several different habitats. The saprotrophic species of *Alternaria* are extensively found in the soil, air, and a variety of other habitats [4, 5]. The plant-pathogenic species of this genus are also ubiquitous agents. *Alternaria* can be also found in normal animal and human skin and conjunctiva [6]. The most common *Alternaria* species include* A. alternata, A. tenuissima, A. arborescens, A. radicina, A. brassicae, A. brassicicola, A.* *malorum,* and* A. infectoria *[7, 8]. 

*Alternaria* *alternata* is one of the most common saprophytes found throughout the world. This species has been clinically associated with asthma, allergic rhinosinusitis, hypersensitivity, oculomycosis, onychomycosis, skin infections, and allergic bronchopulmonary mycosis [9]. *Alternaria* *malorum *causes disseminated phaeohyphomycosis in immuno-competent individuals [8]. *Alternaria* *alternata* is also one of the most important species of *Alternia* that produces AAL toxins, causing many problems for humans and animals and endangering their health [10]. Consumption of foodstuff contaminated with Alternaria toxins is reportedly associated with a raised incidence of esophageal carcinoma in humans [11]. 

Tomato with the scientific name *Solanum lycopersicum* is native to South and Central America, This vegetable crop is now widely planted around the world and grown in greenhouses in cold regions. Iran is considered as one of the major producers of tomatoes. In this regard, in 2013, Iran was the sixth largest tomato producing country in the world with the annual production of 6 million tons [12]. Lycopene found in tomatoes reduces the risk of breast, neck, prostate, and head cancer. In addition, tomato contributes to the health of the immune system through the formation of blood vessels [13-16]. However, this plant is susceptible to fungi due to its thin skin. *Alternaria* is the most common fungus in moldy tomatoes [17,18].

*Alternaria* has over 50 pathogenic and nonpathogenic species that are morphologically similar to each other. The identification of these species at the species level by means of the traditional and morphological methods is time-consuming, difficult, and in most cases, not sufficiently precise [19, 20]. With recent advances in molecular biology, researchers have started using DNA-based molecular techniques and sequencing of genetic regions for the quick and accurate detection of fungi at the species level. 

Moreover, the first step in the study of plant and human pathogens is the identification of fungal species and investigation of their genetic diversity. Therefore, the accurate identification of fungal species provides scientists with useful information about the genetic diversity of the fungal population. Currently, internal transcribed spacer ribosomal RNA (ITS rRNA) is widely used to detect pathogenic fungi at the species level. The study of genetic diversity as a useful tool to understand the biology of pathogenic populations suffices to determine the distribution of pathogens, epidemiologic issues, interactions between host and pathogenic species, and pathogenic variability. Such investigations also lead to the identification of strategies for disease control [21]. 

The inter simple sequence repeat (ISSR) technique includes the utilization of microsatellite sequences as primers in PCR to produce multi-allelic markers. It combines the advantages of microsatellites and amplified fragment length polymorphism (AFLP) markers with the universality of random amplification polymorphic DNA (RAPD). The ISSR is extremely polymorphic and helpful in the study of genetic diversity, phylogeny, molecular characterization, and evolutionary biology [22]. This technique has been effectively used in the investigation of fungal populations, insects, and vertebrates [23].

With this background in mind, the aim of this study was to identify *A. alternata* varieties isolated from tomatoes using morphological and molecular methods based on the sequencing of ITS1 rRNA region. This study was also targeted toward the examination of the genetic diversity of the isolates of this fungus based on the ISSR marker.

## Materials and Methods

***Sampling, cultivation, and identification of fungi***

Sixty tomato samples (in different sizes) with black spots and mildew were collected from the supermarkets of Khorramabad City, Iran, in the winter of 2017. After transferring the tomatoes to the laboratory and washing them with distilled water, their contaminated parts were isolated and then disinfected with 1% sodium hypochlorite superficially. Subsequently, the samples were dried and then cultured on a potato dextrose agar (Merck, Germany) medium plus chloramphenicol (0.05 g/L). The samples were kept at a constant temperature of 25°C for 7 days in an incubator. 

The purification of the isolates was carried out using the single-spore method. The purified isolates were identified based on the described characteristics of *Alternaria* genus (i.e., macroscopic and microscopic characteristics, including size, color, number of longitudinal and transverse walls, presence of surface ornamentation, presence and absence of the tip, length, and size of conidiophore, and presence or absence of secondary conidiophore) and morphological characteristics [24]. In addition, other fungal isolates were identified in the infected tomatoes.

***Molecular identification of Alternaria isolates using the sequencing of ITS1 region***

***DNA extraction***

DNA extraction was carried out using acetyl trimethylammonium bromide (CTAB) with a few changes [25]. The culturing of the fungal isolates was carried out in a medium for 7 days at 25°C. Then, the mycelium was collected and crushed using liquid nitrogen resulting in achieving a homogeneous powder. Afterwards, 700 μl of CTAB buffer (100 mM Tris-HCl, 1.4 M NaCl, 20 mM EDTA, pH of 8.0) containing CTAB [2% (w/v)] was added to 50-100 g of the crushed powder. After mixing, the microtubes were placed at 65°C for 45 min. 

The microtubes were centrifuged for 10 min at 10000×g. Subsequently, 650 μl of the supernatant mixture, along with an equivalent volume of chloroform /Isoamyl alcohol, was centrifuged at 10000×g for 10 min at room temperature. The supernatant phase was isolated, and 0.7 ml of cold isopropyl alcohol was added to the mixture and stored at-20°C for 20 min. The tubes were centrifuged for 5 min at 10000×g. The precipitate containing DNA was washed twice with 70% ethanol and centrifuged at 10000×g for 5 min each time. 

Finally, the pellet was air-dried, and the DNA was resuspended in 30 μl of TE buffer (10 mM Tris–HCl pH of 8, 1 mM EDTA). Nucleic acid concentrations were determined by means of a NanoDrop 1000 spectrophotometer (Thermo Fisher Scientific, Wilmington, DE, USA). In addition, the integrity of each DNA sample was tested on 12 g L−1 agarose gel. Moreover, the quality of the extracted DNA on 1% agarose gel was studied with DAC gel using electrophoresis and DNA safe staining.

***Polymerase chain reaction***

The amplification of the ITS1 region in PCR was accomplished using two primers, namely ITS1 (5'-TCCGTAGGTGAACCTGCGG-3') and ITS4 (TCCTCCGCTTATTGATATGC-3') [26]. The PCR reaction was performed in a Biorad thermocycler (S 1000TM).

**Table 1:** Name and number of scored bands and number of polymorphic bands and polymorphic inter simple sequence repeat primers used in 15 isolates of *Alternaria* *alternenta*

**No**	**Primer name**	**Primer sequence**	**Annealing temperature**	**No. of scored bands**	**No. of polymorphic bands**	**Polymorphism (%)**	**PIC**
1	*ISSR1*	5'-(AG)_8 CC_-3'	49	17	16	94.1	0.44
2	*ISSR2*	5'- (AG)_8 CT_-3'	48	12	12	100	0.43
3	*ISSR3*	5'- (AG)_8 T_-3'	48	9	3	33.3	0.13
4	*ISSR4*	5'- (AG)_8 G_-3'	47	13	13	100	0.42
Total	*-*			51	44	91.5	-

ISSR: inter simple sequence repeat, PIC: polymorphic information content

The PCR included an initial denaturation step of 5 min at 94ºC with 40 cycles of denaturation for 1 min at 94ºC, primer annealing for 45 sec at 53ºC, and primer extension for 90 sec at 72ºC with an initial denaturation at 94ºC for 5 min and a final extension for 10 min at 72ºC. The reaction was performed in a volume of 25 µL containing 2 µL (20 ng/ml) DNA templates, 12.5 µL master mix, and 10 pmol of each primer. 

The PCR products were electrophoresed (for 1 h at 80 V) in 0.8% agarose gel in Tris–borate-EDTA buffer at a pH of 8. The gels were stained with DNA safe stain (10 mg/mL) and observed in a gel documentation system (Alpha Innotech, USA). In the next stage, the products obtained from the PCR of the ITS1 region were sequenced. In addition, the nucleotide sequences obtained using the local BLAST (http://blast.ncbi.nlm.nih.gov/Blast.cgi) were examined and compared with similar sequences in the Gene Bank.

***Genetic variation of the isolated Alternia genes using*** ***inter simple sequence repeat*** ***markers***

To evaluate the genetic diversity of *Alternaria* species, the ISSR marker and six primers were used. Out of the six primers used for genetic diversity, four primers showing more repeatability and polymorphism were used to examine all *A. alternata* species. The primers were selected based on previous studies investigating *Alternaria*. The names, sequences, annealing temperature, and other initiating properties of the primers are shown in Table 1. 

The PCR cycle for the ISSR marker consisted of an initial denaturation step at 94°C for 3 min, followed by 35 cycles of another denaturation at 94°C for 55 sec. Primer annealing was performed for 1 min; in addition, the extension step was carried out at 72°C for 2 min, and the final extension was conducted at 72ºC for 5 min. After completing the PCR steps, the electrophoresis and 1.6% agarose gel were used to detect polymorphism between the specimens. To this end, 5 μl of each sample was added to 2-μl loading buffer. The samples were then loaded in wells, and electrophoresis was performed at 100 V for 150-180 min. 

To determine the size of the components, a marker with the standard bands of 1000 bp and gels were stained with DNA safe stain (10 mg/mL), and the gel was photographed by the DAC gel (Alpha Innotech, USA). In order to investigate polymorphism between the genotypes, the presence and absence of any particular bands were identified with numbers 1 and 0, respectively. Then, for the formation of A raw data matrix, columns and rows were assigned to the genotypes and bands, respectively, and the numbers were entered into the EXCEL software. 

The matrix was used to determine the genetic similarity (using three indices of Jaccard, SM, and Dice), draw a dendrogram, and analyze the balanced components using the NTSYSpc software (version 2.2). After the formation of the similarity matrix, data analysis was performed in the NTSYSpc software. To investigate the polymorphic information content (PIC) of the markers and primers, the PIC parameter (resolution) was applied. The mean value of PIC was used based on the relationship proposed by Pawell et al. [27]. The PIC of each marker was calculated in Excel software using the following equation:

PIC=1-∑f_i_^2^

where *f*_i_ is the frequency of *i*^th^ allele.

For statistical estimation and support of the internal branches of the dendrogram, the grouping of the masses and cultivars was based on the similarity coefficient of SM by the unweighted pair group method with arithmetic mean (UPGMA) method. In addition, tree drawing was performed based on the UPGMA method using the NTSYSpc software.

## Results

***Morphological and molecular identification of isolates extracted from tomatoes***

**Figure 1:**
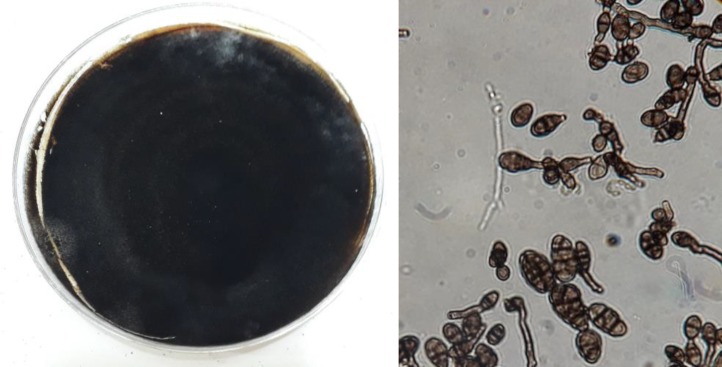
Morphology of colony and conidia isolates of *Alternaria* *alternate*

The species were identified based on their morphological characteristics (i.e., macroscopic and microscopic characteristics), and then compared with the mycological sources. The results revealed that in 60 tomato samples examined, 32 extracted fungal isolates were in conformity with* A. alternate *(n=15), *Penicillium *species (n=5), *Rhizopus *species (n=4), *Fusarium prolifratum* (n=2), *F. oxysporum* (n=2), and *Aspergillus Nigri *complex (n=4; Figure 1).

**Figure 2:**
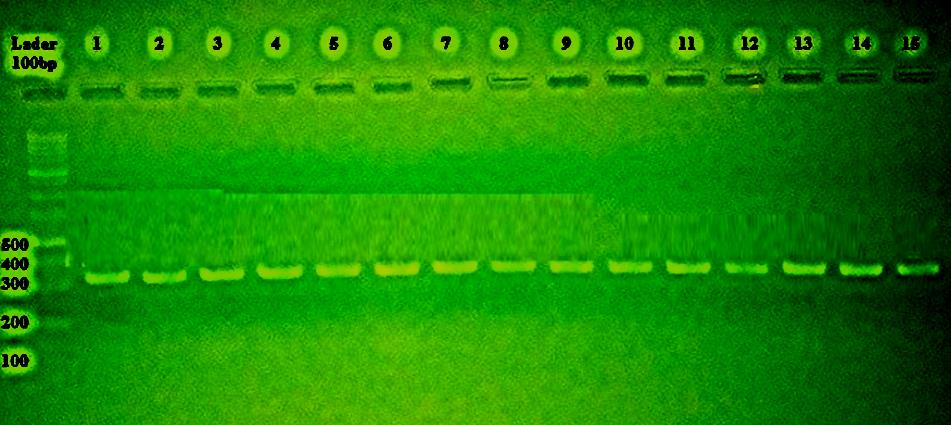
Polymerase chain reaction products of 15 isolates of *Alternaria* *alternenta* with primer ITS1 and 100 bp ladder

In this study, *Alternaria* species were identified using molecular methods based on the sequencing of the ITS-rDNA region of the ribosomal region. The ITS-rDNA region of the isolates was replicated using PCR, and a gene fragment was multiplied to a band size of 360 base pair (bp). Sequences of the ITS-rDNA region of the isolates were compared with those of the NCBI site using the BLAST software. The results showed that the sequence of the extracted isolates had the highest similarity of 99-100% with *A. alternata* species (Figure 2).

**Figure 3:**
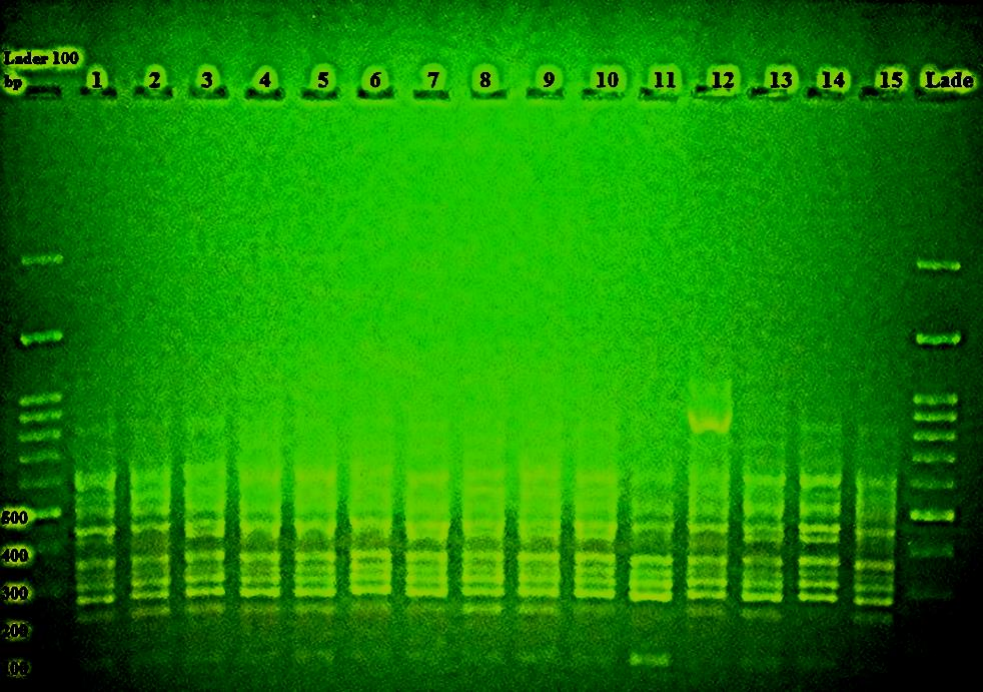
Inter simple sequence repeat-polymerase chain reaction products of 15 isolates of *Alternaria* *alternenta* with primer 2 and 100 bp ladder

***Cluster analysis based on inter simple sequence repeat*** ***markers***

In order to study the genetic diversity of the 15 species of *A. alternata*, the ISSR marker was used; furthermore, the polymorphic bands of four primers were also applied for analysis. All the high-resolution bands were scored at the intervals of 500-1000 pb (Figure 3). The four primers produced a total of 51 bands, 44 (91.5%) cases of which were multi-shaped. Primer 1 with 17 bands had the highest number of bands, whereas primer 3 with 9 bands had the lowest number of bands. The highest and lowest polymorphic bands belonged to the primer 1 with 16 bands (94.1% polymorphism) and the primer 3 with 9 bands (33.3% polymorphism), respectively (Table 1).

**Figure 4:**
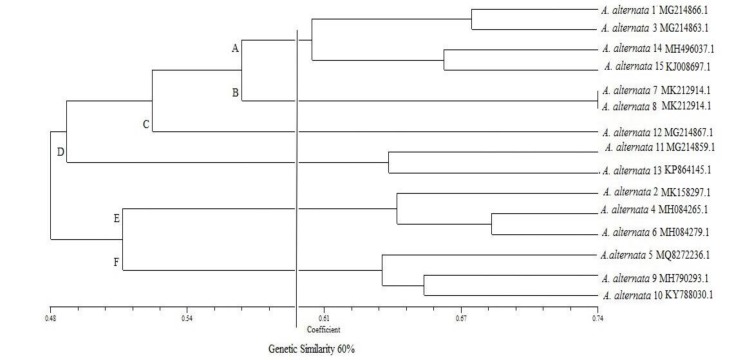
Cluster analysis dendrogram using unweighted pair group method with arithmetic mean method based on the Dice similarity coefficient in inter simple sequence repeat test for 15 *Alternaria* *Alternata* species

The dendrogram obtained from the cluster group of the species was drawn using ISSR on the basis of the Dice similarity coefficient )Figure 4). The correlation coefficient was obtained as 0.66, and similarity range was estimated at 48-74%, based on which six major groups (i.e., A-F) were achieved by cutting the dendrogram into a similarity level of 60%. The obtained groups included Group A (four members), Group B (two members with a similarity of 100%), Group C (one member), Group D (two members), Group E (three members), and Group F (four members).

Group A with four members formed the largest group, whereas Group C with one member was the smallest genetic group. Among the primers, the highest PIC value was 0.44 for the ISSR1 primer, while the lowest PIC value was 0.13 for the ISSR3 primer. The average PIC calculated in this study was 0.35. The high levels of PIC in this study indicated the effectiveness of the primers in differentiating the species.

## Discussion

Organisms causing corruption in foods include molds, yeasts, and bacteria that are always present in the air, water, and soil. Fungi are the most important and common pathogens that infect and destroy a wide variety of fruits and inflict significant economic losses during transportation, storage, and even in markets [28]. Various fungal species, such as *Alternaria*, *Fusarium*, *Penicillium*, *Mucor*, *Rhizopus,* and *Aspergillus,* are known as spoilage organisms [29].

The investigation of the macroscopic and microscopic characteristics of the isolates and comparison of the obtained data with mycological sources resulted in the identification of *A. alternate*, *Penicillium *species, *Rhizopus *species, *F. prolifratum,* *F. oxysporum,* and *Aspergillus Nigri *complex in the tomato samples. In this study, *Alternaia* species that caused spoilage and early blight in the tomatoes were identified using molecular methods based on the sequencing of rDNA in the ITS region and their genetic variation using the ISSR marker. Based on molecular studies, all *Alternaria* species identified by the morphologic method belonged to *A. alternata*. 

The determination of the DNA sequence of rRNA as a fungal identification method, representing a major mutation from traditional methods to molecular methods, is an important step in the identification of taxonomic fungi. In previous studies, various species, including *A. alternata*, *A. niger*, *F. oxysporum*, *Penicillium* species, and *Mucor* species, were isolated from the tomatoes examined after harvest, as well as form tomatoes collected from supermarkets. These results are consistent with those of the present study [30-34]. 

*Alternaria* *alternata* produces several allergens and mycotoxins and has been isolated from a wide range of foods, plants, and animals [35]. This species also results in the complication of human posttraumatic or posttransplant keratomycosis and has growing importance as an opportunistic pathogen in patients with immunodeficiency [35]. Species belonging to the genus *Alternaria,* such as *A. alternata* and *A. tenuissima,* have the ability to produce toxic compounds (e.g., alternariol, alternariol monomethyl ether, altenuen, and tenuazonic acid), which are dangerous for food production. 

Since tomatoes contain a considerable amount of liquid, mycotoxins spread rapidly throughout this vegetable crop, thereby infecting all the parts and making it unsuitable for consumption. According to the literature, *A. alternate* species may be a potential source of mycotoxins that are harmful to human health*.* Therefore,* A. alternata* species isolated in this study are the sources of potent mycotoxins, which are detrimental to health [31, 35-38].

Tomatoes are susceptible to early blight in all the stages of growth [39]. The disease is spreading in a wide range of climatic conditions, especially in semi-warm and warm areas [40]. The warm and semi-warm regions with high relative humidity have favorable environmental conditions for the disease cycle. If the necessary measures are not taken to control the disease, it will damage the plants by eliminating the green bodies [41]. 

According to previous studies, the cause of early blight can be *A. alternata* and *A. solani*. In this regard, Fatih et al. separated *A. alternata* species from rotten tomatoes [32, 42]. The results of the mentioned study showed that all the studied isolates were *A. alternata*, which can be considered a cause of early blight disease. In a number of other studies performed in Iran, *A. alternata* and *A. solani* were isolated from citrus species, including sour orange, chino, and other citrus fruits, as well as potatoes. The early blight disease with *A. solani* is one of the most common potato diseases in potato farmlands. The main hosts of the disease are Solanaceae plants, such as potato, tomatoes, and peppers. 

The ISSR marker was used to study the genetic differences of highly similar species, as well as the genetic differences of fungal species. The ISSR is a selective marker for assessing genetic variation in cocoa, gymnosperm, and even fungi. This marker has been successfully used to estimate the development of intra- and inter-species genetic variation in a wide variety of plant species, including rice, wheat, finger millet, sweet potato, and broadleaf plantain [43, 44].

Various molecular methods, such as RAPD, AFLP, and ISSR, have been used to examine a variety of plant pathogens and fungi at the genome level. The ISSR has important advantages over other molecular methods. The ISSR technique can produce more polymorphic bands and a smaller amount of genomic DNA. This mark does not require the process of enzymatic digestion and ligation, compared to the AFLP technique. Moreover, due to the use of longer oligonucleotide sequences, the ISSR technique is more specific and reproducible, facilitates more stringent annealing conditions in PCR amplification, and has higher levels of polymorphism, compared to the RAPD technique [45]. 

Markers with a high number of alleles are more suitable for studying genetic variation. On the other hand, the high number of alleles represents a high genetic variation in the population under study [46]. In a study conducted by Kale et al. to evaluate the genetic variation of *A. alternate* species, 98% of the alleles (bands) were multiform [47]. In another study carried out by Zafar et al. to investigate the genetic variation of *A. alternate* species, 83.3% of the alleles were multiform [48]. In the present study involving four primers, 51 alleles were observed, 85.81% of which were multiform. 

The PIC amount is one of the important indicators for comparing different markers in terms of differentiation power. The high values of this criterion signify a high polymorphism in the marker that plays a significant role in the differentiation of populations. Qi et al. (2006) reported that primers with a PIC of more than 0.25 were useful indicators for genetic variation. In the present study, the primers ISSR1, ISSR2, and ISSR4 had a PIC of > 0.25, indicating their effectiveness in separating and differentiating the studied populations of *A. alternata*.

The genotypes were classified into six groups based on the similarity coefficient of the Dice and UPGMA method. Accordingly, the isolates in different groups had a similarity range of 48-74%. This high level of genetic diversity can be obtained as a result of a series of evolutionary processes, including mutation, recombination, and migration [49, 50]. Grouping isolates with a distant position can be due to the transfer of the fungus from point to point. Since *Alternaria* spores are dried in the air and can survive for a long time, they are dispersed by wind in different parts of Iran. Therefore, they can contaminate the hosts susceptible to organisms [51].

The results of this study showed the suitability of ISSR marker for genetic variation studies in *Alternaria* species. This marker has been used by other researchers to study the genetic diversity of *Alternaria* species. In contrast to a small number of previous studies, all the isolated varieties of *A. alternata *were reported in this study. It can be argued that in addition to *A. solani*, *A. alternata* is a contributing factor to the early blight spit spot in tomatoes.

Unlike a number of previous studies, in the present study, all the isolates were *A. alternata*, suggesting that, in addition to *A. solani*, *A. alternata* is a contributing factor to the incidence of early blight in tomatoes. The study of genetic diversity offers strategies for controlling this disease. Since *A. alternata* has a strong desire to produce a number of harmful mycotoxins (e.g., as alternariolmonomethyl ether, tenuazonic acid, altenuene, tentoxin, and alternariol), *A. Alternaria* species can be a serious threat to human health through producing toxin in tomato products [52].

## Conclusion

The results of this study indicated the contamination of tomato samples harvested from different regions of Iran with various fungal species, including *Alternaria*. The fungal corruption of these national strategic products induces economic damage. This contamination can pose a potential threat to human health due to the ability of the isolates to produce high levels of *Alternaria* fungal toxins. 

Our results necessitate the implementation of extensive epidemiological studies on the occurrence, dispersal, and genetic variation of these fungi. Such studies could be helpful in the development and utilization of appropriate and effective strategies to control mycotoxin and fungal contamination in human food, as well as in animal and agricultural products.
